# Exploring the Dynamic Role of Bacterial Etiology in Complicated Urinary Tract Infections

**DOI:** 10.3390/medicina59091686

**Published:** 2023-09-20

**Authors:** Mădălin Guliciuc, Daniel Porav-Hodade, Raul Mihailov, Laura-Florentina Rebegea, Septimiu Toader Voidazan, Veronica Maria Ghirca, Adrian Cornel Maier, Monica Marinescu, Dorel Firescu

**Affiliations:** 1 Clinical Emergency County Hospital “Sf. Ap. Andrei”, 800578 Galati, Romania; guliciuc.madalin@gmail.com (M.G.); raulmihailov@yahoo.com (R.M.); laura_rebegea@yahoo.com (L.-F.R.); 2 Faculty of Medicine and Pharmacy, Dunarea de Jos University, 800008 Galati, Romania; maier23adrian@yahoo.com (A.C.M.); dorelfirescu@yahoo.com (D.F.); 3 Faculty of Medicine and Pharmacy, “George Emil Palade” University of Medicine, Pharmacy, Science and Technology of Targu Mures, 540139 Târgu Mures, Romania; septi_26_07@yahoo.com (S.T.V.); veronica.ghirca@yahoo.com (V.M.G.); 4 Emergency Military Hospital Galati, 800150 Galati, Romania; fixmonica@yahoo.com

**Keywords:** urosepsis, complicated urinary tract infections, multiple drug-resistant bacteria, antibiotics

## Abstract

*Background and Objectives*. Numerous studies have been conducted to explore the epidemiological characteristics of urinary tract infections (UTI) and sepsis. However, there is still a lack of relevant bacteriological features and prognostic information regarding urosepsis based on bacteriological etiology. The current study aims to evaluate the bacterial etiology of complicated UTI (cUTI) and bacterial resistance to antibiotics and whether they present an intrinsic risk of developing urosepsis. *Materials and Methods*. A retrospective study was performed that included 102 patients who were diagnosed with cUTI and admitted to the urology department of the “Sfântul Apostol Andrei” County Emergency Clinical Hospital (GCH) from September 2019 to May 2022. *Results.* A considerable number of patients, n = 41 (40.2%), were diagnosed with multi drug-resistant (MDR) infection. *Escherichia coli* (*E. coli*) was identified as the prevailing pathogen, accounting for 51 patients. *Klebsiella* manifested itself as the subsequent causative agent in 27 instances. The presence of *Enterococcus* spp. infection was documented in 13 patients, whereas *Pseudomonas* emerged as the etiological perpetrator in the clinical context of 8 patients. The current study found a substantial prevalence of resistance to first-line antibiotics. The overall resistance rate was 74.5% for penicillin, 58.82% for trimethoprim–sulfamethoxazole and 49% for fluoroquinolones; cephalosporin resistance displayed an inverse correlation with antibiotic generation with fourth-generation cephalosporins exhibiting a resistance rate of 24.5%, and first-generation cephalosporins demonstrating a resistance rate of 35.29%. *Conclusions*. Age, comorbidities and indwelling urinary catheters are risk factors for developing MDR infections. While the intrinsic characteristics of the causative bacterial agent in cUTI may not be a risk factor for developing urosepsis, they can contribute to increased mortality risk. For empiric antibiotic treatment in patients with cUTI who are at a high risk of developing urosepsis and experiencing a potentially unfavorable clinical course, broad-spectrum antibiotic therapy is recommended. This may include antibiotics, such as amikacin, tigecycline, carbapenems and piperacillin–tazobactam.

## 1. Introduction

Urinary tract infections (UTI) can present clinically in a variety of ways from asymptomatic bacteriuria to septic shock. The severity of UTI largely depends on the host response. Numerous studies have been conducted to explore the epidemiological characteristics of UTI and sepsis. However, there is still a lack of relevant bacteriological features and prognostic information regarding urosepsis based on bacteriological etiology [1].

Complicated UTI (cUTI) occur in a person in whom it is considered that systemic, morphological or functional abnormalities of the urinary tract make the infection more difficult to eradicate [2].

Urosepsis is a life-threatening organ dysfunction caused by an abnormal response to an infection originating from the urinary tract or male genitalia. This aberrant response is mediated by a cascade of immunological events. Due to the high mortality rate associated with urosepsis, prompt therapeutic intervention is essential [3].

Standard definitions for various categories of drug-resistant bacteria have been proposed by an international assembly of department heads from specialized forums, such as the European Center for Disease Prevention and Control, the Office of Infectious Diseases, the Department of Health and Human Services, the Center for Disease Prevention and Control and the Division of Epidemiology at Tel Aviv Sourasky Medical Center. These definitions categorize drug-resistant bacteria as follows:

- Multi drug resistant (MDR): Referring to strains that are not susceptible to one or more antibiotic agents in three or more antimicrobial categories.
- “Extensive” or “extremely” drug resistant” (XDR): Describing strains that are not susceptible to one or more antibiotic agents in all but two or fewer antimicrobial classes.
- “Pan drug resistant” (PDR): Designating strains that are not susceptible to any antimicrobial agents listed [4,5].

Although the guidelines of the European Association of Urology (EAU) provide valuable guidance on the diagnosis and treatment of various UTI, there are still significant differences in the spectrum of pathogens, bacterial antibiotic resistance and the risk of progression to urosepsis globally [6].

The current study aims to evaluate the bacterial etiology of cUTI and bacterial resistance to antibiotics and whether they present an intrinsic risk of developing urosepsis.

## 2. Materials and Methods

A retrospective study was performed that included patients who were diagnosed with cUTI and admitted to the urology department of the GCH, a general hospital in Galati, Romania. The study period spanned from September 2019 to May 2022. GCH, an 800-bed facility, is situated in Galati City, accommodating a resident population of 250,000 individuals. The hospital caters to the healthcare needs of the Galati County region, which comprises a total population of 450,000 people.

Ethical approval for this study was granted by the ethics committee of GCH, Galati, Romania, under the reference number 24363/2021.

### 2.1. Patients Selection

Given the exhaustive nature of the definition regarding cUTI [2], for the purpose of facilitating comprehension and practical implementation, we shall expound upon the most prevalent risk factors associated with the pathogenesis of cUTI. The risk factors associated with cUTI encompass both systemic factors, such as male gender, diabetes and immunosuppression, as well as local factors, including urinary tract obstruction, intracavitary foreign bodies, urinary diversion, chronic urinary retention, vesicoureteral reflux, recent urinary tract instrumentation and long-term catheterization [2]. The inclusion criteria were a confirmed UTI based on urine culture and the presence of local or systemic risk factors associated with cUTI.

We applied the following exclusion criteria: patients diagnosed using only clinical symptoms, aged below 18 years, pregnancy, history of kidney transplantation, hemodialysis or peritoneal dialysis and missing data. Given that our study period overlaps with the COVID-19 pandemic, it is important to state that, from our cohort, 2 patients were concurrently diagnosed with COVID-19 infection and cUTI. As these patients were subsequently transferred to other healthcare units designated for treating SARS-CoV-2, they were excluded from our study.

### 2.2. Data Collection

Prior to admission, a comprehensive clinical evaluation was conducted, including various parameters: heart rate, blood pressure, respiratory rate, PaO_2_, temperature and Glasgow Coma Scale. Subsequent to admission, blood and urine samples were obtained in accordance with the International Safety Standards [7]. Upon hospital admission, assessments were conducted for complete blood count (CBC), total bilirubin, creatinine and procalcitonin (PCT) levels.

Demographic information, clinical manifestations, laboratory findings and diagnostic evaluations were documented. A thorough examination of the patients’ medical records was carried out, and pertinent clinical and biological data were extracted. To assess the comorbidities that may predispose the patient to an immunocompromised status, we utilized the Charlson Comorbidity Index (CCI) [8].

The urine collection procedure adhered to the International Safety Standards. Sterile receptacles were used to collect the urine samples, which were subsequently employed for microbial culturing on laboratory-prepared Columbia sheep agar and lactose agar. In specific instances, the Chapman medium was utilized for the identification of *Staphylococcus* spp. Bacterial incubation was conducted for 24 h at 37 °C. Only pure microbial cultures were considered, and a urine density of 105 CFU/mL was deemed indicative of the presence of Gram-negative bacilli. The Vitek automatic microbiology system was employed to ascertain colony density in CFU/mL [9]. Manual techniques were employed as a contingency measure in the case of technical malfunctions with the equipment. Bacteriuria was determined by applying the urine specimen onto Petri dishes followed by calculation using the formula X = N × D × 1/inoculated volume, where X represents the CFU/mL count, N denotes the number of colonies observed on the Petri dish, and D stands for the dilution factor [10].

The identification of bacteria relied on a morphological assessment of the colonies as well as biochemical characteristics, such as lactose fermentation, indole production, urease activity, lysine decarboxylase activity and hydrogen sulfide production for enterobacteria [11]. In cases where distinguishing between different types of enterobacteria posed challenges, the Vitek automatic system was employed for identification [9].

The disk diffusion technique, conducted in accordance with the Clinical Laboratory Standards Institute (CLSI) guidelines, was used to determine the antimicrobial susceptibility of each bacterial strain. This involved utilizing pure microbial cultures to prepare a 0.5 McFarland inoculum in a saline solution. After applying the standardized inoculum onto Petri dishes, eight antibiotic discs were placed equidistantly from each other and from the edge of the dish, while a ninth antibiotic disc was positioned at the center of the plate.

Following overnight incubation, the diameters of the inhibition zones were measured using the disk diffusion technique (Kirby–Bauer method). The obtained results were compared to the CLSI standards to ascertain the resistance and susceptibility patterns of each pathogenic agent [12,13].

### 2.3. Patient Grouping

In order to assess the impact of bacterial resistance on the clinical outcomes of patients with cUTI, including the risk of urosepsis and mortality, we divided the patients into two different groups. The first group, referred to as the non-MDR group, comprises patients who exhibit resistance to fewer than three classes of antibiotics. Conversely, the second group, known as the MDR group, consists of patients who demonstrate resistance to three or more classes of antibiotics.

Based on the clinical and paraclinical presentation of the patients, as well as adhering to current definitions, we categorized the patients into two groups: those diagnosed with cUTI and those with urosepsis. The patients diagnosed with urosepsis were those who presented upon admission or during the course of hospitalization cUTI proven with urine culture and systemic inflammatory response syndrome (SIRS).

SIRS corresponds to the presence of two of the following criteria:

- Fever, temperature above 38 °C or hypothermia below 36 °C.
- Tachycardia—over 90/min.
- Tachypnea—over 20 breaths/min or partial pressure of carbon dioxide in the arterial blood (PaCO^2^) < 32 mm Hg.
- Leukocytosis > 12,000/mm^3^ or leukopenia < 4000/mm^3^ or the presence of immature cells in the periphery < 10% [14].

The cohort of patients who progressed to septic shock and subsequently expired during hospitalization was categorized into a distinct group, referred to as the deceased group.

### 2.4. Statistical Analysis

The data were considered as nominal or quantitative variables. The nominal variables were characterized using frequencies. The quantitative variables were tested for normality of distribution using the Kolmogorov–Smirnov test and were characterized by median and minimum–maximum or by mean and standard deviation (SD), when appropriate. A chi-square test was used in order to compare the frequencies of the nominal variables. The quantitative variables were compared using the Student *t* test or Mann–Whitney U test, when appropriate.

The level of statistical significance was set at *p* < 0.05. The statistical analysis was performed using SPSS for Windows version 23.0 (SPSS, Inc., Chicago, IL, USA).

## 3. Results

The current investigation involved the enrollment of a cohort that consisted of 102 participants. Out of the total number of patients, 33 were females (33.35%), while 37 (36.27%) originated from rural areas. The mean age of the included patients was determined to be 60.78 ± 15.99 years.

A total of 64 patients (62.75%) developed urosepsis, and the rest were classified as the cUTI group.

Out of the entire cohort of patients, a total of 14 individuals, representing a proportion of 13.72%, expired during their inpatient stay.

A considerable number of patients, 41 (40.2%), were diagnosed with MDR infection, reflecting the complexity and importance of this clinical condition.

From the total number of patients enrolled in our study, a subset of 32 individuals, accounting for 31.37%, was identified as chronic urinary catheter carrier, including urethral–vesical catheters, cystostomies, ureteral splints and stents as well as nephrostomies. This observation underscores the presence of a significant population requiring specific monitoring and management.

**Figure 1:**
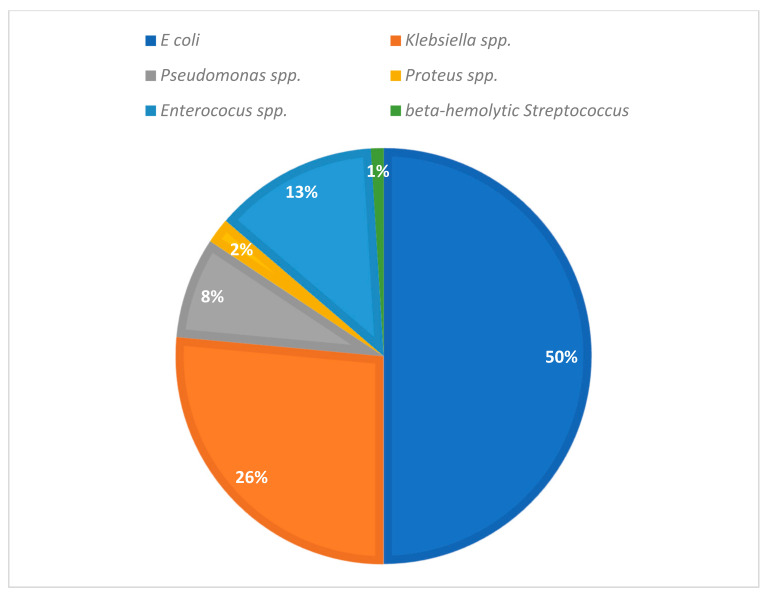
Bacteriological etiology.

From a bacteriological etiological standpoint, *Escherichia coli* (*E. coli*) was identified as the prevailing pathogen, accounting for 51 patients and constituting a notable 50% of the cases under investigation. Notably, *Klebsiella* manifested itself as the subsequent causative agent in 27 instances, representing a significant proportion (26.47%). The presence of *Enterococcus* spp. infection was documented in 13 patients (12.74%), whereas *Pseudomonas* emerged as the etiological perpetrator in the clinical context of eight patients (7.8%). It is worth mentioning that an infection caused by *Proteus* was reported in two patients, while an additional patient developed an infection due to *beta-hemolytic Streptococcus* (Figure 1).

**Figure 2:**
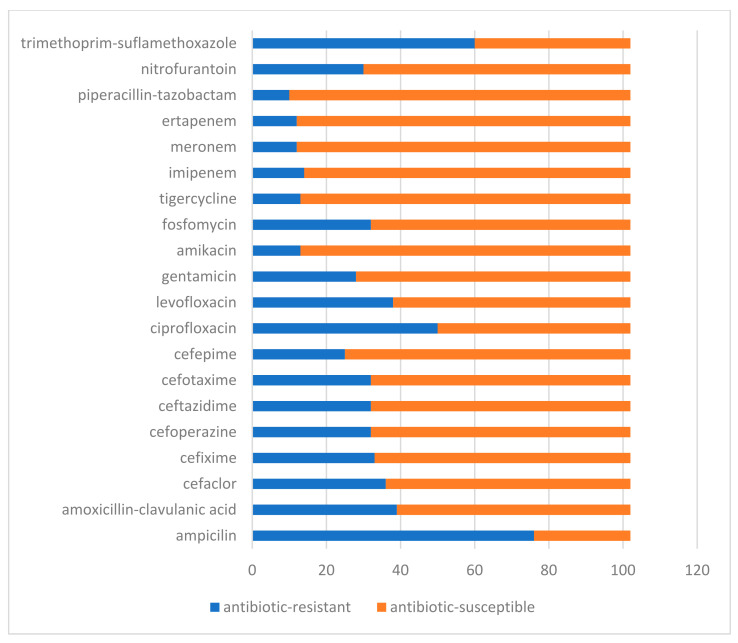
Bacterial antibiotic resistance.

When examining the antibiotic resistance transcending the specific microbial type, a noteworthy observation emerges. Of particular concern is the escalating resistance encountered towards the penicillins with ampicillin exhibiting a strikingly high resistance rate of 74.5%. Similarly, the trimethoprim–sulfamethoxazole combination elicits considerable resistance with bacteria demonstrating a resistance prevalence of 58.82%. Fluoroquinolones, namely ciprofloxacin and levofloxacin, encounter a substantial hurdle, displaying resistance rates of 49% and 37.25%, respectively. Notably, cephalosporins portray a consistent resistance pattern with a gradual declining trend concerning successive generations. Specifically, the first-generation cephalosporins exhibit a resistance rate of 35.29% followed by 32.35% for the second generation, 31.37% for the third generation, and a relatively lower resistance rate of 24.5% for the fourth generation represented by Cefepime. The emergence of resistance is also encountered with amikacin and tigecycline, affecting 12.75% of the bacterial population. In the realm of carbapenems, a similar resistance profile is observed with meropenem and ertapenem demonstrating a parallel resistance rate of 11.76%. However, it is worth highlighting that piperacillin–tazobactam emerges as a potent therapeutic resource, as only 9.8% of the studied bacterial strains exhibited resistance to this combination (Figure 2).

**Figure 3:**
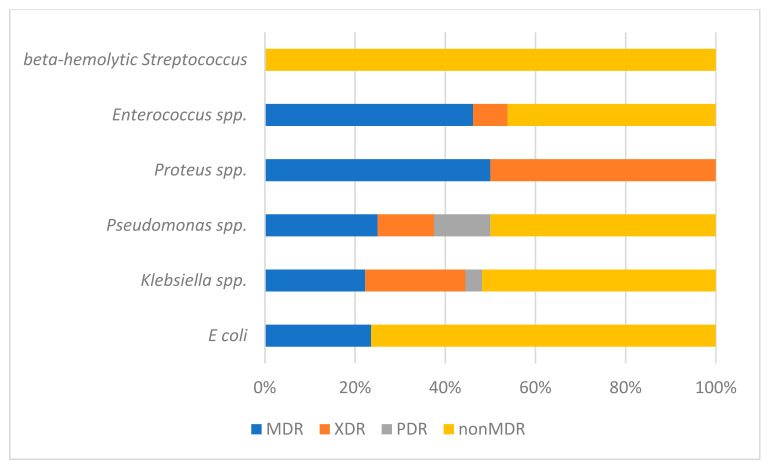
MDR status according to bacterial etiology.

Upon individual examination of the antibiotic resistance profile for each bacterial species, distinctive patterns become evident. Particularly noteworthy is the observation within the realm of *E coli*, where a subset of 12 strains, representing 23.53% of the cases (Figure 3), demonstrates the highly concerning MDR phenotype. Regarding the patients diagnosed with *Klebsiella* infection, a significant proportion of 13 individuals, accounting for 48.14% of the analyzed cohort, displayed the worrisome development of MDR infection. Among these patients, a notable subset of six individuals, constituting 22.22% of the total, progressed further to acquire an XDR infection, while a solitary patient (3.7%) exhibited the alarming PDR phenotype. In the case of *Enterococcus* strains, an important finding emerges with seven isolates, representing 53.84% of the cohort, demonstrating the MDR phenotype (Figure 3). As for *Pseudomonas* infections, 50% are classified as MDR (Figure 3) with one strain identified as XDR and another strain identified as PDR. Our study encompassed only two patients who developed *Proteus* infection. All of these bacterial strains were identified as MDR (Figure 3) with one of them classified as XDR.

The chi-square test was employed to highlight the correlation between the presence of MDR infection and the existence of indwelling urinary catheters, resulting in a *p*-value of 0.002 that signifies a significant statistical interdependence. For the purpose of evaluating if MDR infection represents an intrinsic risk factor for mortality, the chi-square test was used, which generated a *p*-value of 0.048, establishing the statistical significance of this association. Utilizing the same test, we examined whether there is a correlation between MDR infection and the risk of developing urosepsis. The resulting *p*-value of 0.199 suggests no significant interdependence between the two variables. In the same vein, it was demonstrated that there are no correlations between gender and urban or rural environment and the risk of developing MDR infection (Table 1).

By employing the Mann–Whitney U test, we assessed the interdependence between comorbidities, represented by CCI, and MDR infection, resulting in a *p*-value below 0.001 and, thereby, confirming this hypothesis. Using the Student t test, we analyzed the correlation between age and the risk of developing MDR infection, returning a *p*-value below 0.001 and, thus, demonstrating this interdependence (Table 1). Likewise, the same test was applied to evaluate the correlation between age and the existence of indwelling urinary catheters, yielding a *p* < 0.001 that indicates a strong correlation between these two variables.

To highlight whether there is a correlation between the bacterial etiology of cUTI and the risk of developing urosepsis, we employed the chi-squared test. We conducted an analysis on the bacteria that were most frequently encountered. No statistically significant correlation was found in the case of *E. coli* (*p* = 0.41), *Klebsiella* (*p* = 0.06), or *Enterococcus* (*p* = 0.60). *Pseudomonas* infection proved to be a protective factor against the development of urosepsis (*p* = 0.021, OR = 0.171) (Table 2).

To investigate whether cUTI pose a risk of being MDR based on bacterial etiology, we utilized the chi-squared test. An analysis on the bacteria that were most frequently encountered was conducted. *E. coli* infection proved to be a protective factor against antibiotic resistance (*p* = 0.001, OR 0.233). The other bacterial etiologies did not show a statistically significant association with MDR infection (Table 3).

## 4. Discussion

cUTI and urosepsis are urological disorders that exhibit an escalating prevalence rate. Considering the increased frequency of the risk factors associated with the occurrence of this condition, we are obliged to take proactive measures. Our endeavor encompasses the exploration of novel approaches aimed at the early identification and optimal management of this pathological entity with the ultimate goal of averting the deleterious course towards urosepsis and the potentially adverse evolution of these conditions. Moreover, it is crucial to address the issue of antibiotic misuse, as it contributes to the emergence of MDR bacterial phenotypes that, in turn, pose an inherent treatment challenge.

In our study, the mean age of the patients was 60.78 ± 15.99 years. This finding substantiates that cUTI are a condition with an increased prevalence associated with advancing age. We consider that the comorbidities commonly associated with older age actually serve as risk factors for the development of cUTI as statistically demonstrated using the Spearman rho test (*p* < 0.001). Furthermore, there is a significant age-related dependence regarding the risk of developing an MDR infection (*p* < 0.001). The aforementioned observation gains further credence from the significant statistical correlation between advancing age and the utilization of enduring urinary drainage apparatus (*p* < 0.001) as well as the noteworthy association between urinary devices and the heightened susceptibility to MDR infections (*p* = 0.002). Mostafa et al. concluded in their paper that the increasing use of indwelling catheters led to a serious number of complications with infection being the most common. The pathogens responsible for UTI form biofilms on medical device surfaces, enabling them to evade host defenses and develop resistance to antimicrobial agents [15]. In a 2016 study that involved a cohort of 585 patients diagnosed with urosepsis, a notable association was discovered between the usage of permanent urinary catheters, the presence of comorbidities and advanced age and an escalated incidence of MDR infections, ultimately resulting in an augmented vulnerability to the development of septic shock [16].

The observed prevalence of MDR infections in our study (40.2%) slightly exceeds the rates reported in the existing literature. This disparity can be attributed primarily to the distinctive attributes of our study sample, which consisted of elderly patients with multiple comorbidities and an extensive medicalization history [17]. *E. coli* strains isolated from urosepsis patients have a lower prevalence of genetic characteristics that are phenotypically translated into virulence and are less likely to originate from a uropathogenic clone than strains isolated from patients with uncomplicated UTI. The organisms isolated from cUTI and urosepsis tend to be more antibiotic-resistant than the strains isolated in uncomplicated UTI [18].

In our study, we did not find evidence of a statistically significant association (*p* = 0.199) between MDR infection and the risk of developing urosepsis. This finding aligns with the conclusions drawn by Shaw et al. who conducted a separate study and determined that host factors rather than the specific microorganisms or patterns of antimicrobial resistance primarily influenced the occurrence of urosepsis [19]. Nevertheless, MDR infection has been established as an autonomous risk factor for mortality (*p* = 0.048). It is recognized that patients afflicted with MDR infection frequently exhibit a higher burden of comorbidities and an extensive medical history. Moreover, there exists a strong correlation between MDR infection and the provision of inadequate empirical antibiotic therapy. While some studies highlight MDR infection as an independent risk factor for mortality, others emphasize its role as a risk factor for inappropriate antibiotic therapy. The latter, in turn, is identified as an independent risk factor for mortality [20,21].

Within the scope of our study, it was determined that Gram-negative bacteria constituted the predominant etiological agents in the pathogenesis. Among these, *E. coli* accounted for the highest prevalence, representing 50% of the cases, followed by *Klebsiella* at a proportion of 26.47%. *Enterococcus* and *Pseudomonas* were also identified, comprising 12.74% and 7.8% of the cases, respectively. The literature indicates that Gram-negative bacteria were identified as the predominant causative agents, attributing to the majority of cUIT cases. The distribution of Gram-negative bacteria within the cohort analyzed by Wagenlehner et al. in 2007 was as follows: *E. coli* accounted for 50% of cases, while *Proteus* spp., *Enterobacter* and *Klebsiella* collectively contributed to 15% of cases. *Pseudomonas aeruginosa* was identified as the causative factor in 5% of cases [22]. The microbial landscape of cUTI exhibits heterogeneity, encompassing a diverse array of Gram-negative and Gram-positive bacterial species. This spectrum of bacteria may demonstrate geographic variations, temporal fluctuations and intra-institutional disparities even within the same healthcare facility [23,24,25]. It is of paramount importance for every healthcare facility to establish a comprehensive surveillance system to accurately document UTI, specifically focusing on complicated and nosocomial UTI. This meticulous record-keeping enables a more individualized and tailored approach in addressing such infections.

When evaluating whether bacterial etiology might inherently pose a risk of developing urosepsis, the most commonly encountered bacteria did not exhibit statistical significance. *Pseudomonas*, on the other hand, emerged as a protective factor against urosepsis development (*p* = 0.021, OR = 0.171). This indicates that cUTI caused by this specific etiological agent have a lower risk of evolving into urosepsis. However, given that our cohort comprises only eight cases (7.8%) of *Pseudomonas* infections, we cannot extrapolate this result confidently. Further investigations on larger cohorts will be necessary to validate this finding.

Regarding antibiotic resistance, our study focused on patients with cUTI and urosepsis, many of whom had significant comorbidities, advanced age and indwelling urinary catheters. We found a substantial prevalence of resistance to first-line antibiotics. Specifically, penicillins exhibited an overall resistance rate of 74.5% with *Klebsiella* strains demonstrating intrinsic resistance to ampicillin (100%). Moreover, we identified elevated resistance rates of 58.82% for trimethoprim–sulfamethoxazole and 49% for fluoroquinolones including ciprofloxacin, while levofloxacin displayed a resistance rate of 37.25%. Notably, cephalosporin resistance displayed an inverse correlation with antibiotic generation with fourth-generation cephalosporins exhibiting a resistance rate of 24.5% and first-generation cephalosporins demonstrating a resistance rate of 35.29%. According to our study findings, in order to achieve the desired therapeutic effect using the principle of escalation in antibiotic therapy for patients with urosepsis, the aforementioned antibiotics should be excluded with the potential exception of fourth-generation cephalosporins. Antibiotics that have been shown to exhibit low resistance rates include amikacin, tigecycline (12.75%), carbapenems (11.76%) and piperacillin–tazobactam (9.8%). These agents should be considered when treating a patient with urosepsis and a potentially unfavorable prognosis.

A study conducted in Romania in 2018 that involved 916 patients diagnosed with UTI identified *E. coli* as the primary etiological agent with a prevalence of 42.9% followed by Enterococcus faecalis with a prevalence of 21.17% and *Klebsiella* spp. with a prevalence of 18.66%. This research revealed antibiotic resistance rates for Levofloxacin exceeding 30% in the case of *E. coli* and over 40% for Enterococcus. Additionally, it was found that Klebsiella strains had developed significant resistance to carbapenems and aminoglycosides with a prevalence of over 10%. Although the reported resistance rates in that study are alarming, it should be noted that they are lower compared to those found in our study. However, it is important to emphasize that the Romania study included all UTI, while our research focused exclusively on analyzing cUTI. This distinction reinforces the idea that the same empirical antibiotic treatment protocol should not be applied for cUTI as in the case of uncomplicated UTI [26,27]. Bichoff’s research identifies specific risk factors linked to antibiotic resistance in UTI. These risk factors align with the conditions that increase an individual’s susceptibility to cUTI. The study suggests that, in cases where these risk factors are absent, cephalosporins are a suitable choice for empirical therapy. However, for patients presenting with these risk factors, piperacillin–tazobactam outperforms fluoroquinolones, cephalosporins or gentamicin. This study underscores the importance of local surveillance of resistance rates and risk factors to optimize empirical therapy within a particular geographic context [28]. Similarly, in a study conducted by Lee et al., it was concluded that, when managing patients with UTI who meet the criteria for critical sepsis upon initial presentation, it is advisable to consider the empirical prescription of broad-spectrum antibiotics capable of addressing potential patterns of drug resistance. Such antibiotics may include tigecycline, carbapenems or fourth-generation cephalosporins [15]. In 2019, Jiang et al. published a retrospective study that analyzed 94 patients diagnosed with urosepsis. The primary etiological agent identified was *E. coli* (64.62%) followed by *Klebsiella* spp. (21.84%). The study demonstrated a resistance rate exceeding 80% for penicillins, first- to third-generation cephalosporins and quinolones with a susceptibility of 50% for aminoglycosides and 100% for carbapenems [29]. The global prevalence of infections in urology is a study conducted by Tandogdu and colleagues that encompasses patients admitted to urology departments worldwide. The study assesses healthcare-associated infections and their risk of progressing to urosepsis. The most frequently identified pathogen is *E. coli* (43%) followed by *Enterococcus* spp., *Pseudomonas aeruginosa* and *Klebsiella* spp. An MDR rate of 45% has been reported for Enterobacteriaceae. The only class of antibiotics exhibiting a resistance rate lower than 10% was found to be carbapenems [27]. Given the fact that MDR bacteria are more frequently encountered as etiological agents of cUTI and urosepsis, including carbapenem resistance, Chen enumerates as potential therapeutic resources, in this regard, polymyxins, fosfomycin, tigecycline, nitrofurantoin, linezolid and daptomycin. However, it is imperative to underscore that this conclusion necessitates further studies [30].

To comprehensively understand the antibiotic resistance profiles of each individual bacterial species, substantial proportions of MDR cases are observed in *Proteus* (100%), *Enterococcus* (53.86%) and *Pseudomonas* (50%). However, given the relatively low occurrence of these bacteria in our study, it is not feasible to draw meaningful comparisons and determine their impact on the progression of cUTI within our specific geographic area. To enhance understanding, this study would benefit from the inclusion of a larger and more diverse database, involving a greater number of subjects collected prospectively over an extended period. By analyzing whether the bacterial species involved in cUTI carry an individual risk of being MDR, we identified that *E. coli* has a lower risk of being MDR (*p* = 0.001, OR 0.233). This indicates that *E. coli* infections are more likely to be community-acquired and not associated with healthcare settings [18]. The same conclusion is drawn from the results analyzed and published in 2018 by the European Centre for Disease Prevention and Control (ECDC) regarding antibiotic resistance in the case of *E. coli* (e.g., 22.8% for third-generation cephalosporins and 0% for carbapenems) in Romania [31]. This report also highlights the MDR rate in infections caused by *Klebsilla* spp. (e.g., 67.3% for third-generation cephalosporins and 20.5% for carbapenems) in our country along with the future risk of lacking therapeutic solutions for managing infections caused by this pathogenic agent [31]. Our study also raises the concerning aspect that resides in the fact that *Klebsiella*, as a prevalent pathogenic agent occurring in more than a quarter of cases, displays a substantial MDR rate of 48.14%. Among these cases, a noteworthy subset of 22.22% exhibits XDR characteristics, while an additional 3.7% demonstrates the alarming PDR phenotype. Klebsiella is the second most frequent etiological agent but exhibits significant resistance to first-line antibiotics, as previously demonstrated by Mishra in India [32] and Petca in Romania [26]. It is imperative to conduct further investigations to ascertain the potential association of this strain with healthcare-related infections and implement proactive strategies aimed at curtailing its transmission dynamics.

## 5. Conclusions

Age, comorbidities and indwelling urinary catheters are risk factors for developing MDR infections. While the intrinsic characteristics of the causative bacterial agent in cUTI may not be a risk factor for developing urosepsis, they can contribute to increased mortality risk. Thus, MDR bacteria are associated with higher mortality rates among the patients in our study. For empiric antibiotic treatment in patients with cUTI who are at a high risk of developing urosepsis and experiencing a potentially unfavorable clinical course, broad-spectrum antibiotic therapy is recommended. This may include antibiotics, such as amikacin, tigecycline, carbapenems and piperacillin–tazobactam.

## Figures and Tables

**Table 1:** Bivariate analysis of variables according to MDR status.

	All Patients (n = 102)	Non-MDR (n = 61)	MDR (n = 41)	*p* Value
Age (years), mean +/− SD	60.78 =/− 15.996	55.07 +/− 15.616	69.29 +/− 12.498	0.0001 **
Environment, urban (n, %)	65 (63.7)	42 (68.9)	23 (56.1)	0.18 *
Sex, male (n, %)	69 (67.6)	37 (60.7)	32 (78.0)	*p* = 0.066 *
CCI (median–min–max)	7-0-13	4-0-13	8-1-13	*p* = 0.004 ***
Deceased (n, %)	14 (13.7)	5 (8.2)	9 (22.0)	*p* = 0.048 *
Indwelling urinary catheters (n, %)	32 (31.4)	12 (19.7)	20 (48.8)	*p* = 0.002 *
Urosepsis (n, %)	64 (64.7)	36 (59.0)	26 (63.4)	*p* = 0.199 *

* Chi-square test; ** Student *t* test; *** Mann–Whitney U test.

**Table 2:** Bivariate analysis of bacterial species according to urosepsis status.

	All Patients (n = 102)	cUTI (n = 38)	Urosepsis (n = 64)	*p* Value *	OR (CI 95%)
*E coli* (n, %)	51 (50)	21 (55.3)	30 (46.9)	0.41	0.71 (32–160)
*Klebsiella* spp. (n, %)	27 (26.5)	6 (15.8)	21 (32.8)	0.06	2.6 (94–71.9)
*Enterococcus* spp. (n, %)	13 (12.7)	4 (10.5)	9 (14.1)	0.60	1.39 (39–486)
*Pseudomonas* spp. (n, %)	8 (7.8)	6 (15.8)	2 (3.1)	0.021	0.172 (33–90)

* Chi-square test; OR-odds ratio; CI-confidence interval.

**Table 3:** Bivariate analysis of bacterial species according to MDR status.

	All Patients (n = 102)	Non-MDR (n = 61)	MDR (n = 41)	*p* Value *	OR (CI 95%)
*E coli* (n, %)	51 (50)	39 (62.9)	12 (29.3)	0.001	0.233 (10.0–54.0)
*Klebsiella* spp. (n, %)	27 (26.5)	12 (19.7)	15 (36.6)	0.058	2.356 (9.6–57.7)
*Enterococcus* spp. (n, %)	13 (12.7)	6 (9.8)	7 (17.1)	0.283	1.887 (58.5–60.88)
*Pseudomonas* spp. (n, %)	8 (7.8)	3 (4.9%)	5 (12.2%)	0.180	2.685 (60.5–119.2)

* Chi-square test; OR-odds ratio; CI-confidence interval.

## Data Availability

The database used for statistical analysis, Excel worksheets, as well as descriptive and analytical statistical analysis, can be found in the Supplementary Materials section.

## Supplementary Materials

The following supporting information can be downloaded at: https://www.mdpi.com/article/10.3390/medicina59091686/s1.
