# Supplementary material for: Exploring the Dynamic Role of Bacterial Etiology in Complicated Urinary Tract Infections
Source: Medicina (Kaunas). 2023 Sep 20;59(9):1686. doi: 10.3390/medicina59091686 (PMC10538164; doi:10.3390/medicina59091686)
Supplement: Supplementary file 1 [file medicina-59-01686-s001.zip › stat.pdf]

Teste de tip chi pătrat
Vei folosi tabele de genul

Table S1

	**All patients (102)**	**nonMDR 61**	**MDR 41**	**p value**
Age (years), mean+/-SD	70.46+/-12.3	73.75+/-9.56	68.27+/-13.5	0.0001**
Mediu U (n, %)	65 (63.7)	42 (68.9)	23 (56.1)	0.18*
Sex, male (n, %)				
qSOFA				
0				
1				
2				
3				
CCI (median-min-max)				
Decont (median-min-max)				
Zile spit (median-min-max)				
Decedat (n, %)				
Dispozitiv (n, %)				
Lot dg (n, %)				
	**All patients (102)**	**cUIT 38**	**Urosepsis 64**	**p value**
Age (years), mean+/-SD				
Mediu U (n, %)	65 (63.7)	28 (73.7)	37 (57.8)	0.10*
Sex, male (n, %)				
qSOFA				
0				
1				
2				
3				
CCI (median-min-max)				
Decont (median-min-max)				
Zile spit (median-min-max)				
Decedat (n, %)				
Dispozitiv (n, %)				
Lot MDR (n, %)				

Crosstab

0.18			lotMDR 0	lotMDR 1	Total
mediu	R	Count	19	18	37
		% within mediu	51.4%	48.6%	100.0%
		% within lotMDR	31.1%	43.9%	36.3%
	U	Count	42	23	65
		% within mediu	64.6%	35.4%	100.0%
		% within lotMDR	68.9%	56.1%	63.7%
Total		Count	61	41	102
		% within mediu	59.8%	40.2%	100.0%
		% within lotMDR	100.0%	100.0%	100.0%

mediu * lotdg

Crosstab

P-0.10			lotdg 0	lotdg 1	Total
mediu	R	Count	10	27	37
		% within mediu	27.0%	73.0%	100.0%
		% within lotdg	26.3%	42.2%	36.3%
	U	Count	28	37	65
		% within mediu	43.1%	56.9%	100.0%
		% within lotdg	73.7%	57.8%	63.7%
Total		Count	38	64	102
		% within mediu	37.3%	62.7%	100.0%
		% within lotdg	100.0%	100.0%	100.0%

sex * lotMDR

Crosstab

p-0.066			lotMDR 0	lotMDR 1	Total
sex	0	Count	24	9	33
		% within sex	72.7%	27.3%	100.0%

	% within lotMDR	39.3%	22.0%	32.4%
1	Count	37	32	69
	% within sex	53.6%	46.4%	100.0%
	% within lotMDR	60.7%	78.0%	67.6%
Total	Count	61	41	102
	% within sex	59.8%	40.2%	100.0%
	% within lotMDR	100.0%	100.0%	100.0%

sex * lotdg

Crosstab

p-0.89			lotdg		Total
			0	1	
sex	0	Count	12	21	33
		% within sex	36.4%	63.6%	100.0%
		% within lotdg	31.6%	32.8%	32.4%
	1	Count	26	43	69
		% within sex	37.7%	62.3%	100.0%
		% within lotdg	68.4%	67.2%	67.6%
Total		Count	38	64	102
		% within sex	37.3%	62.7%	100.0%
		% within lotdg	100.0%	100.0%	100.0%

qSOFA * lotMDR

Crosstab

p-0.70			lotMDR		Total
			0	1	
qSOFA	0	Count	25	17	42
		% within qSOFA	59.5%	40.5%	100.0%
		% within lotMDR	41.0%	41.5%	41.2%
	1	Count	19	9	28
		% within qSOFA	67.9%	32.1%	100.0%
		% within lotMDR	31.1%	22.0%	27.5%
	2	Count	13	11	24

		% within qSOFA	54.2%	45.8%	100.0%
		% within lotMDR	21.3%	26.8%	23.5%
		Count	4	4	8
	3	% within qSOFA	50.0%	50.0%	100.0%
		% within lotMDR	6.6%	9.8%	7.8%
		Count	61	41	102
Total		% within qSOFA	59.8%	40.2%	100.0%
		% within lotMDR	100.0%	100.0%	100.0%

qSOFA * lotdg

Crosstab

p-0.0001 semnificativ			lotdg		Total
			0	1	
qSOFA	0	Count	28	14	42
		% within qSOFA	66.7%	33.3%	100.0%
		% within lotdg	73.7%	21.9%	41.2%
	1	Count	7	21	28
		% within qSOFA	25.0%	75.0%	100.0%
		% within lotdg	18.4%	32.8%	27.5%
	2	Count	3	21	24
		% within qSOFA	12.5%	87.5%	100.0%
		% within lotdg	7.9%	32.8%	23.5%
	3	Count	0	8	8
		% within qSOFA	0.0%	100.0%	100.0%
		% within lotdg	0.0%	12.5%	7.8%
Total		Count	38	64	102
		% within qSOFA	37.3%	62.7%	100.0%
		% within lotdg	100.0%	100.0%	100.0%

decedat * lotMDR

Crosstab

p-0.048 semnifiariv			lotMDR		Total
			0	1	
decedat	0	Count	56	32	88

		% within decedat	63.6%	36.4%	100.0%
		% within lotMDR	91.8%	78.0%	86.3%
		Count	5	9	14
	1	% within decedat	35.7%	64.3%	100.0%
		% within lotMDR	8.2%	22.0%	13.7%
		Count	61	41	102
Total		% within decedat	59.8%	40.2%	100.0%
		% within lotMDR	100.0%	100.0%	100.0%

decedat * lotdg

Crosstab

p-0.002 semnificativ			lotdg		Total
			0	1	
decedat	0	Count	38	50	88
		% within decedat	43.2%	56.8%	100.0%
		% within lotdg	100.0%	78.1%	86.3%
	1	Count	0	14	14
		% within decedat	0.0%	100.0%	100.0%
		% within lotdg	0.0%	21.9%	13.7%
Total		Count	38	64	102
		% within decedat	37.3%	62.7%	100.0%
		% within lotdg	100.0%	100.0%	100.0%

dispozitiv * lotMDR

Crosstab

p-0.002 semnificativ			lotMDR		Total
			0	1	
dispozitiv	0	Count	49	21	70
		% within dispozitiv	70.0%	30.0%	100.0%
		% within lotMDR	80.3%	51.2%	68.6%
	1	Count	12	20	32
		% within dispozitiv	37.5%	62.5%	100.0%
		% within lotMDR	19.7%	48.8%	31.4%
Total		Count	61	41	102

	% within dispozitiv	59.8%	40.2%	100.0%
	% within lotMDR	100.0%	100.0%	100.0%

dispozitiv * lotdg

Crosstab

p-0.009 semnificativ			lotdg		Total
			0	1	
dispozitiv	0	Count	32	38	70
		% within dispozitiv	45.7%	54.3%	100.0%
		% within lotdg	84.2%	59.4%	68.6%
	1	Count	6	26	32
		% within dispozitiv	18.8%	81.2%	100.0%
		% within lotdg	15.8%	40.6%	31.4%
Total		Count	38	64	102
		% within dispozitiv	37.3%	62.7%	100.0%
		% within lotdg	100.0%	100.0%	100.0%

Ce este cu rosu introduci in tabele

Vârsta CCI decont zilespit * lotdg

lotdg		Vârsta	CCI	decont	zilespit
0	Mean	54.05	4.61	3934.32	6.16
	Std. Deviation	17.820	3.997	3330.097	2.955
	Median	54.50	4.00	2723.50	6.00
	Minimum	19	0	459	1
	Maximum	84	12	11568	13
1	Mean	64.78	7.13	12422.87	15.56
	Std. Deviation	13.422	3.557	12097.067	11.769
	Median	67.50	7.50	8400.00	11.00
	Minimum	33	0	834	2
	Maximum	91	13	69230	63
Total	Mean	60.78	6.19	9260.47	12.06
	Std. Deviation	15.996	3.904	10599.694	10.511
	Median	62.00	7.00	6415.00	9.00
	Minimum	19	0	459	1
	Maximum	91	13	69230	63

Vârsta CCI decont zilespit * lotMDR

lotMDR		Vârsta	CCI	decont	zilespit
0	Mean	55.07	4.79	8655.11	11.15
	Std. Deviation	15.616	3.760	9160.160	9.738
	Median	55.00	4.00	6397.00	8.00
	Minimum	19	0	459	1
	Maximum	85	13	41715	50
1	Mean	69.29	8.27	10161.12	13.41
	Std. Deviation	12.498	3.139	12507.578	11.556
	Median	71.00	8.00	6433.00	10.00
	Minimum	43	1	620	3
	Maximum	91	13	69230	63
Total	Mean	60.78	6.19	9260.47	12.06
	Std. Deviation	15.996	3.904	10599.694	10.511
	Median	62.00	7.00	6415.00	9.00
	Minimum	19	0	459	1
	Maximum	91	13	69230	63

Group Statistics

Teste Student	lotdg	N	Mean	Std. Deviation	P value
Vârsta	1	64	64.78	13.422	0.001
	0	38	54.05	17.820	

Test Statistics[a]

	CCI	decont	zilespit
Mann-Whitney U	802.000	542.000	433.500
Wilcoxon W	1543.000	1283.000	1174.500
Z	-2.881	-4.665	-5.427
P value	.004	.0001	.0001

a. Grouping Variable: lotdg

Group Statistics

Teste Student	lotMDR	N	Mean	Std. Deviation	P value
Vârsta	1	41	69.29	12.498	0.0001

0	61	55.07	15.616	

Test Statistics[a]

	CCI	decont	zilespit
Mann-Whitney U	598.000	1179.000	1062.500
Wilcoxon W	2489.000	3070.000	2953.500
Z	-4.478	-.488	-1.286
P value	.0001	.626	.199

a. Grouping Variable: lotMDR

Statistica multivariate, regresie logistică multivariata

Variabila dependenta este lotdg, celelalte sunt variabilele independente, Urosepsisul este direct și pozitiv influentat de Qsofa si zilele prelungite de spitalizare, si culmea negativ influentat de MDR

Table S2

		B	S.E.	Wald	Sig.	Exp(B)	95% C.I.for EXP(B)	
							Lower	Upper
Step 1[a]	Vârsta	.025	.033	.583	.445	1.026	.961	1.095
	sex	.477	.727	.430	.512	1.611	.388	6.696
	qSOFA	1.445	.513	7.935	.005	4.242	1.552	11.592
	CCI	.093	.141	.432	.511	1.097	.832	1.447
	decont	.000	.000	1.091	.296	1.000	1.000	1.000
	zilespit	.427	.138	9.528	.002	1.532	1.169	2.009
	decedat	19.012	8642.515	.000	.998	1.032	.945	1.965
	lotMDR	-1.712	.765	5.005	.025	.181	.040	.809

Variabila dependenta este lotMDR, celelalte sunt variabilele independente, MDR este direct și pozitiv influentat de varsta si zilele prelungite de spitalizare

Table S3

Variables in the Equation

		B	S.E.	Wald	df	Sig.	Exp(B)	95% C.I.for EXP(B)	
								Lower	Upper
Step 1[a]	Vârsta	.064	.028	5.266	1	.022	1.067	1.009	1.127
	sex	.966	.608	2.521	1	.112	2.627	.797	8.657

	qSOFA	-.100	.354	.080	1	.777	.905	.452	1.810
	CCI	.141	.109	1.654	1	.198	1.151	.929	1.426
	decont	.000	.000	2.820	1	.093	1.000	1.000	1.000
	zilespit	.109	.057	3.623	1	.047	1.115	1.007	1.248
	decedat	.894	.912	.960	1	.327	2.444	.409	14.606
	lotdg	-1.230	.657	3.502	1	.061	.292	.081	1.060

a. Variable(s) entered on step 1: Vârsta, sex, qSOFA, CCI, decont, zilespit, decedat, lotdg.

Analiza statistica

Data were considered as nominal or quantitative variables. Nominal variables were characterized using frequencies. Quantitative variables were tested for normality of distribution using Kolmogorov-Smirnov test and were characterized by median and minimum-maximum or by mean and standard deviation (SD), when appropriate. A chi-square test was used in order to compare the frequencies of nominal variables. Quantitative variables were compared using t test, Mann-Whitney test, when appropriate. Multivariate analysis was carried out using logistic regressions. We used as dependent variable the lot dg and lot MDR. We included as independent variables: age, gender, qSOFA, CCI,……… .

The level of statistical significance was set at $p<0.05$. Statistical analysis was performed using SPSS for Windows version 23.0 (SPSS, Inc., Chicago, IL).
